# Compatibility of early natural enemy introductions in commercial pepper and tomato greenhouses with repeated pesticide applications

**DOI:** 10.1111/1744-7917.12723

**Published:** 2019-09-17

**Authors:** Beatriz Dáder, Ignacio Colomer, Ángeles Adán, Pilar Medina, Elisa Viñuela

**Affiliations:** ^1^ Unidad de Protección de Cultivos, Escuela Técnica Superior de Ingeniería Agronómica, Alimentaria y de Biosistemas (ETSIAAB) Universidad Politécnica de Madrid (UPM) Madrid Spain; ^2^ Departamento de Ingeniería Rural, Escuela Superior de Ingeniería Universidad de Almería Almería Spain

**Keywords:** *Amblyseius swirskii*, chemical control; *Frankliniella occidentalis*, *Nesidiocoris tenuis*, *Orius laevigatus*, *Tuta absoluta*

## Abstract

Successful integrated pest management in protected crops implies an evaluation of the compatibility of pesticides and natural enemies (NE), as control strategies that only rely on one tactic can fail when pest populations exceed NE activity or pests become resistant to pesticides. Nowadays in Almería (Spain), growers release NE prior to transplanting or early in the crop cycle to favor their settlement before pest arrival because this improves biocontrol efficacy, although it extends pesticide exposure periods. The purpose of this research was to evaluate the compatibility of two applications of pesticides with key NE in 2‐year trials inside tomato and sweet pepper commercial greenhouses: *Nesidiocoris tenuis* (Reuter) (Hemiptera: Miridae), *Orius laevigatus* (Say) (Hemiptera: Anthocoridae) and *Amblyseius swirskii* (Athias‐Henriot) (Acari: Phytoseiidae). In tomato, flubendiamide and chlorantraniliprole (IOBC category 1) were compatible with *N. tenuis*, but chlorpyrifos‐methyl and spinosad (IOBC categories 2–3), which effectively reduced *Tuta absoluta* (Meyrick) (Lepidoptera: Gelechiidae) density, compromised its predatory activity. In sweet pepper, chlorantraniliprole (IOBC category 1) was the only pesticide compatible with *O. laevigatus* while chlorantraniliprole, emamectin benzoate, spirotetramat and pymetrozine were harmless (IOBC category 1) to *Amblyseius swirskii*, and sulfoxaflor slightly harmful (IOBC category 2) to this phytoseiid predator.

## Introduction

The region Almería, in Southeastern Spain, has developed an intensive protected crop industry (48 000 ha), which has been the driving force of the socioeconomic development in recent decades. The two main crops are sweet pepper and tomato (MAPAMA, 2018a). Currently, one of the most aggressive pests affecting tomato crops is the South American tomato pinworm *Tuta absoluta* (Meyrick) (Lepidoptera: Gelechiidae), present in European, African, Asian, and South American tomato‐producing areas (Biondi *et al*., 2018; Mansour *et al*., 2018; Han *et al*., 2019). This invasive pest favors secondary pathogen infections and reduces the photosynthetic activity of the host plant and thus its production (Desneux *et al*., 2010; Tropea Garzia *et al*., 2010). The Western flower thrips, *Frankliniella occidentalis* (Pergande) (Thysanoptera: Thripidae) and the tobacco whitefly, *Bemisia tabaci* (Gennadius) (Hemiptera: Aleyrodidae) are other important pests for both tomato and pepper. They cause economic losses by feeding when populations reach high levels and interfere with crop physiological processes, by transmission of plant viruses and by production of vast amounts of honeydew, which promotes sooty mold growth (De Barro, 2011; CABI, 2018).

Successful Integrated Pest Management (IPM) systems, mandatory in the European Union (EU) since the implementation of the directive on the sustainable use of pesticides, 2009/128/EC (OJEU, 2009), came into effect in 2014 along with a variety of control measures (Ehler, 2006). IPM programs based on biological control had been practicing in the region for many years since campaign 2005–2006, undoubtedly in parallel with the increase in crop area under this strategy; from 129 ha in the 2005–2006 campaign to 26 590 ha in 2016–2017 (Biocolor, 2018). In the region of Almería, due to the high presence of insecticide resistance (Elbert & Nauen, 2000; Espinosa *et al*., 2002; Bielza *et al*., 2007; Fernández *et al*., 2009; Grávalos *et al*., 2014), virtually all greenhouses follow IPM programs primarily based on biological strategies occasionally supported with selective chemical treatments (Robledo *et al*., 2009; Glass & Egea González, 2012). Several polyphagous predators are commercialized to control sweet pepper and tomato pests. The phytoseiid mite *Amblyseius swirskii* (Athias‐Henriot) (Acari: Phytoseiidae) is the most widely used NE in the region to control *B. tabaci*, and it is also a very efficient predator of *F. occidentalis* even at low pest densities (Chow *et al*., 2008; Colomer *et al*., 2011; Amor *et al*., 2012; Calvo *et al*., 2012a). Two other interesting NE available are the anthocorid *Orius laevigatus* (Say) (Hemiptera: Anthocoridae), widely used in inoculative releases against *F. occidentalis* (Sánchez *et al*., 2000; Brodsgaard, 2004), and the generalist zoophytophagous mirid *Nesidiocoris tenuis* (Reuter) (Hemiptera: Miridae). The latter species often appears spontaneously in the Mediterranean region (Calvo *et al*., 2012a), feeds on thrips, mites, aphids, leafminers, and whiteflies (Sánchez *et al*., 2009; Pérez‐Hedo & Urbaneja, 2015; Perdikis & Arvaniti, 2016; Bouagga *et al*., 2018), and can regulate *T. absoluta* by feeding on eggs and young larvae (Urbaneja *et al*., 2012; Biondi *et al*., 2018).

Pest control strategies that rely on one approach seem easy and adequate yet often fail when pest populations exceed NE activity or pests become resistant to pesticides (Glass & Egea González, 2012). Therefore, despite the ability of NE to control several pests with simultaneous outbreaks in the crop, chemical treatments are often sometimes needed to maintain key and secondary pest populations under economic thresholds and the ascertaining of their compatibility is crucial for success. Thus, toxicity and sublethal effects of pesticides on NE of horticultural pests has been extensively studied under laboratory or semifield conditions (Amor *et al*., 2012; Bengochea *et al*., 2012; Abraham *et al*., 2013; Döker *et al*., 2014; Garzón *et al*., 2015; Maia *et al*., 2016; Wanumen *et al*., 2016; De Bortoli *et al*., 2017; Fernández *et al*., 2017, Madbouni *et al*., 2017). In general, much less information is available on the compatibility of novel pesticides and NE inside commercial greenhouses. Some formulations and concentrations of methoxyfenozide, chlorantraniliprole, flonicamid, spiromesifen, and sulfur have been found to be compatible with *A. swirskii* and *O. laevigatus* (Bielza *et al*., 2009; Colomer *et al*., 2011; Gázquez *et al*., 2011; Gradish *et al*., 2011). Emamectin benzoate was only compatible in semifield when applied before the NE introduction (Amor *et al*., 2012).

Nowadays, the presence of NE in crops in sufficient numbers before the arrival of pests is considered determinant for biological control efficacy (Sánchez *et al*., 2014; Bouagga *et al*., 2018) and NE producers offer long duration products, which are well suited to a preventive approach (Koppert, 2018). Therefore, *A. swirskii* or *O. laevigatus* are introduced in crops shortly after transplanting while *N. tenuis* is released in the nursery so when the plants are transplanted they already contain the eggs in their tissue, which accelerates NE colonization and establishment (Calvo *et al*., 2012a,b). However, the early introduction of NE in crops makes its coincidence with any necessary pesticide applications unavoidable; thus NE are subjected to the action of the applied active substances for longer time periods.

The objective of this research was to determine whether the preplant introduction of *N. tenuis* in tomato and early introductions of *A. swirskii* and *O. laevigatus* in sweet pepper commercial multispan greenhouses could be compatible with repeated applications of some of the most frequently used pesticides in the region of Almería.

## Materials and methods

### Study sites and growing conditions

The trials took place in 2016 and 2017 in El Ejido (Almería, Spain) inside representative tomato and sweet pepper commercial multispan plastic greenhouses of 1–1.5 hectares, with 100 mm artificial sand mulch soil, and drip irrigation and fertilization carried out according to standard practices in the area.

Crops were transplanted from nursery seedlings and handled according to good standard agricultural practices. *Solanum lycopersicum* L. var. Delizia in 2016 and var. Rambo in 2017 were transplanted on August 18–19 and August 22–23, respectively, at a density of 1.5 plants per 0.4 m^2^. *Capsicum annuum* L. var. Palermo was transplanted on August 23–25, 2016, at a density of 1.5 plants per 0.5 m^2^.

### Natural enemies

Natural enemies were introduced at the initial commercial rates recommended by the manufacturer (Koppert España SL, La Mojonera, Almería, Spain; Koppert, 2018). The mirid *N. tenuis* (Nesibug^®^, 500 mL bottles, 500 adults + nymphs with vermiculite) was released in the nursery at a rate of 0.5 individual per plant, 5 d before transplanting, using Dibox^®^ application boxes on the top leaves of the tomato seedlings with three leaves from the main stem unfolded (BBCH 13; Meier, 2001). To ensure NE survival from its introduction until the arrival of the pests, Entofood^®^ (*Ephestia kuehniella* Zeller eggs + *Artemia* sp. Cysten; 500 mL bottles) was added at a rate of 60 g per row every 7 d, three to four times, using a Mini‐Airbug^®^ device. In sweet pepper, the two NE were early released, 19 d after transplanting, when the first inflorescence opened (BBCH 61; Meier, 2001). The anthocorid bug *O. laevigatus* (Thripor‐L^®^, 100 mL bottles, 2000 adults + nymphs mixed with vermiculite and buckwheat husks) was released at a rate of 4 individuals per m^2^ using Dibox^®^ boxes on the top leaves. The predatory mite *A. swirskii* (Swirskii‐mite LD^®^, paper sachets with 125 mobile forms mixed with wheat bran, various developmental stages of the mite *Carpoglyphus lactis* L. and other acari as a food source) was released at a rate of 75 individuals per m^2^ by hanging the sachets on the middle leaves.

### Pesticide application

Pesticides with distinct modes of action (FRAC, 2018; IRAC, 2018) were selected among the most frequently used inside Almería greenhouses for the control of key pests, based on lack of information on the compatibility with NE according to field technicians and manufacturers. All active ingredients (a.i.) were registered in the EU (MAPAMA, 2018b) and tested at their maximum field recommended concentrations (MFRC) in accordance with the Spanish registration (Table 1). Following regular farmer's pest control practices, plots in both crops were sprayed with the broad‐spectrum and systemic fungicide tebuconazole in order to control *Botrytis cinerea* Pers.:Fr (anamorphic form) powdery mildew and other fungal diseases.

**Table 1:** Active ingredients (a.i.), trademark names, IRAC/FRAC modes of action, applied concentrations, target pests, and crops

Active ingredient	Commercial trademark names in Spain	IRAC/FRAC† modes of action	MFRC‡(g a.i./ha)	Pests	Crop
Chlorantraniliprole	Altacor^®^	Ryanodine receptor modulator	40	Caterpillars	Sweet pepper,
	DuPont, Madrid			*T. absoluta*	tomato
Chlorpyrifos‐methyl	Reldan E^®^	Acetylcholinesterase (AChE) inhibitor	900	Caterpillars thrips	Tomato
	Dow AgroSciences, Madrid				
Emamectin benzoate	Affirm^®^ 0.85% SG	Glutamate‐gated chloride channel (GluCl) allosteric modulator	12.75	Caterpillars	Sweet pepper, tomato
	Syngenta Agro S.A., Madrid			*T. absoluta*	
Flubendiamide	Fenos^®^ 24% WG	Ryanodine receptor	90	Caterpillars	Tomato
	Bayer Cropscience S.L., Valencia	modulator		*T. absoluta*	
Metaflumizone	Alverde^®^ 24% SC	Voltage‐dependent sodium channel blocker	240	Caterpillars,	Tomato
	BASF Española S.L., Madrid			*T. absoluta*	
Pymetrozine	Plenum^®^	Chordotonal organ TRPV (transient receptor potential vanilloid) channel modulator		Aphids, whiteflies	Sweet pepper
	Syngenta, Madrid				
Spinosad	Spintor^®^	Nicotinic acetylcholine receptors (nAChRs)	110	Caterpillars, thrips	Tomato
	Dow AgroSciences, Madrid				
Spirotetramat	Movento^®^ 15% SC	Inhibitor of acetyl CoA	75	Aphids, whiteflies, scales	Sweet pepper
	Bayer Cropscience S.L., Valencia	carboxylase			
Sulfoxaflor	Isoclast^®^	nAChR agonist	24	Aphids, whiteflies	Sweet pepper
	Dow AgroSciences, Madrid				
Tebuconazole§	Folicur^®^ 25 WG	C14‐demethylase in sterol biosynthesis	600	Fungal diseases	Sweet pepper, tomato
	Bayer Cropscience S.L., Valencia		1500		

^†^ IRAC = Insecticide Resistance Action Committee; FRAC = Fungicide Resistance Action Committee.

^‡^ Maximum field recommended concentration.

^§^ Control, following farmers’ regular practices.

Two trials with two pesticide applications were carried out in tomato (September 30 and October 11 in 2016; October 11 and 21 in 2017) and one trial in sweet pepper (October 6 and 18 in 2016), with same or different pesticides based on farmers’ interest. Because in our greenhouses the soil is artificial, a strip trial design (Milliken & Johnson, 1984) with five to six insecticide treatments and two controls randomly distributed was performed in an area per treatment of 72–100 m^2^ with six rows of plants 2 m apart oriented North (N)–South (S). Treatment distribution from East to West was as follows, in tomato 2016: chlorantraniliprole, flubendiamide, control 1, metaflumizone, chlorpyrifos‐methyl, spinosad, control 2 and emamectin benzoate; in tomato 2017: flubendiamide, metaflumizone, control 1, chlorpyrifos‐methyl, spinosad, control 2 and emamectin benzoate; in sweet pepper 2016: emamectin benzoate, control 1, sulfoxaflor, spirotetramat, control 2, pymetrozine and chlorantraniliprole.

In every treatment, four replicates (18–25 m^2^) from the open sides covered with anti‐pest nets (N) to the central corridor (S) were established because pest distribution could be biased, which in turn could have an influence on the natural enemy density.

### Sampling

Direct visual data collection of mobile forms of pests and natural enemies was carried out weekly, with the aid of a small magnifier (6 ×) from 9:00 a.m. to 2:00 p.m., on plants located in the two central rows of each replicate and treatment to avoid spray drift contamination from adjacent pesticides, and on the preferred loci of each species. In tomato, in 2016 we examined 60 leaves per treatment (15 per replicate) on the upper part of the plants to monitor *N. tenuis* and *T. absoluta*; in 2017, only 30 leaves per treatment (7–8 per replicate) because populations were very homogenous. On sweet pepper, we examined 60 leaves per treatment (15 per replicate) from the middle part of flowering plants to monitor mobile forms of *A. swirskii*, and 40 flowers per treatment (10 per replicate) for *O. laevigatus* and *F. occidentalis*.

### Statistical analysis

Statistical analyses of data (presented as mean ± SEM) were carried out using IBM Statistics SPSS v.23.0 package (IBM Corp., 2015). The weighted mean numbers of insects per replicate (*n* = 4; dependent variable), very homogeneous among samples of the same replicate, were used for statistical analyses (Crawley, 2013). Initially, homogeneity of control plots in every crop and year (*P* < 0.05) was studied with a Student's *t*‐test or a nonparametric Mann–Whitney *U*‐test when neither raw nor transformed data followed criteria, and because no statistically significant differences were obtained, an average control was calculated in every case (Colomer *et al*., 2011). The data were analyzed with a Lineal Mixed–effect Model (LMM) (*P* < 0.05), very appropriate for our approach (Wang & Goonewardene, 2004; Crawley, 2013). The lowest value of Akaike's Information Criterion (AIC) was used to select the best covariance structure in each model (Wang & Goonewardene, 2004). The significance of the effects was determined by the *F*‐test statistic and the estimated marginal means were compared with the LSD test. Because pesticides changed with the crop and year, separately analyses were carried out. The different insect species and pesticides were considered as fixed factors and the sampling dates as a repeated measures factor. Based on the NE density on the last sampling date compared to the control, the pesticide effect was categorized according to the four IOBC (International Organization for Biological and integrated Control) toxicity categories for semifield conditions because NE were released in the crops: 1 = harmless: <25% mortality; 2 = slightly harmful: 25%–50% mortality; 3 = moderately harmful: 51%–75% mortality; or 4 = harmful: >75% mortality (Hassan, 1994).

## Results

Pests *T. absoluta* in tomato and *F. occidentalis* in sweet pepper infested the crops. The whitefly *B. tabaci* was not present in either of the crops. The predatory mite *Balaustium hernandezi* von Heyden (Acari: Erythraeidae) was also found in sweet pepper but at a very low density; thus it was not considered. The numbers of pests and NE were found to be homogeneous among the two control replicates of each trial (Table 2). When pooling insect density data of the same year and crop together, there were statistically significant interactions among factors (Table 3); therefore, we proceeded to analyze each insect separately in every crop each year.

**Table 2:** Mean ± SEM population density of *N. tenuis*, *Orius laevigatus*, *Amblyseius swirskii*, *Tuta absoluta*, and *Frankliniella occidentalis* per leaf in two control plots in tomato and sweet pepper with corresponding sampling dates

Tomato 2016	Sept 29	Oct 6	Oct 13	Oct 20	Oct 27			
*N. tenuis*								
C1	3.55 ± 0.23	4.55 ± 0.22	4.88 ± 0.17	5.70 ± 0.23	6.00 ± 0.17			
C2	3.63 ± 0.18	4.95 ± 0.17	5.00 ± 0.13	5.88 ± 0.24	5.95 ± 0.17			
	*T* = −0.258	*U* = 3.500	*U* = 6.000	*U* = 6.000	*U* = 7.500			
	df = 6	Z = −1.307	Z = −0.584	Z = −0.577	Z = −0.146			
	*P* = 0.805	*P* = 0.191	*P* = 0.559	*P* = 0.564	*P* = 0.884			
*T. absoluta*								
C1	0.15 ± 0.09	0.15 ± 0.03	0.13 ± 0.06	0.15 ± 0.06	0.10 ± 0.04			
C2	0.20 ± 0.04	0.18 ± 0.05	0.20 ± 0.04	0.10 ± 0.04	0.10 ± 0.07			
	*U* = 5.000	*U* = 7.000	*U* = 4.500	*U* = 6.000	*U* = 7.000			
	Z = −0.0.893	Z = −0.319	Z = −1.049	Z = −0.599	Z = −0.303			
	*P* = 0.486	*P* = 0.886	*P* = 0.343	*P* = 0.686	*P* = 0.886			

Tomato 2017	Oct 10	Oct 17	Oct 24	Oct 31	Nov 7			
*N. tenuis*								
C1	3.90 ± 0.23	4.93 ± 0.24	5.23 ± 0.14	6.05 ± 0.24	6.33 ± 0.21			
C2	3.98 ± 0.19	5.25 ± 0.19	5.33 ± 0.11	5.93 ± 0.23	6.33 ± 0.17			
	*t* = −0.249	*U* = 4.500	*U* = 6.500	*t* = −0.374	*t* = −0.000			
	df = 6	Z = −1.016	Z = −0.447	df = 6	df = 6			
	*P* = 0.812	*P* = 0.309	*P* = 0.655	*P* = 0.721	*P* = 1.000			
*T. absoluta*								
C1	1.33 ± 0.06	1.33 ± 0.05	1.25 ± 0.09	1.20 ± 0.11	1.23 ± 0.09			
C2	1.35 ± 0.06	1.30 ± 0.04	1.35 ± 0.06	1.20 ± 0.07	0.68 ± 0.17			
	*t* = −0.277	*U* = 6.500	*U* = 5.500	*U* = 7.500	*U* = 0.500			
	df = 6	*Z* = −0.458	*Z* = −0.744	*Z* = −0.149	*Z* = −2.178			
	*P* = 0.791	*P* = 0.686	*P* = 0.486	*P* = 0.886	*P* = 0.029			

Sweet pepper 2016	Oct 5	Oct 12	Oct 19	Oct 26	Nov 3	Nov 9	Nov 16	Nov 23
*O. laevigatus*								
C1	1.03 ± 0.30	0.73 ± 0.13	0.70 ± 0.04	0.88 ± 0.13	0.78 ± 0.15	0.63 ± 0.12	0.95 ± 0.16	0.75 ± 0.16
C2	1.05 ± 0.13	1.13 ± 0.19	1.18 ± 0.05	1.35 ± 0.09	1.33 ± 0.11	0.85 ± 0.12	0.83 ± 0.08	0.93 ± 0.06
	*t* = −0.077	*t* = −1.712	*t* = −7.592	*t* = −3.017	*t* = −2.874	*t* = −1.342	*t* = −0.724	*t* = −1.043
	df = 6	df = 6	df = 6	df = 6	df = 6	df = 6	df = 6	df = 6
	*P* = 0.941	*P* = 0.138	*P* < 0.001	*P* = 0.023	*P* = 0.028	*P* = 0.228	*P* = 0.496	*P* = 0.337
*A. swirskii*								
C1	3.35 ± 0.34	3.25 ± 0.43	2.85 ± 0.34	2.58 ± 0.39	2.80 ± 0.15	2.30 ± 0.19	2.38 ± 0.11	2.75 ± 0.13
C2	3.63 ± 0.64	2.88 ± 0.52	3.08 ± 0.06	2.25 ± 0.23	2.53 ± 0.19	2.45 ± 0.21	2.90 ± 0.36	2.93 ± 0.13
	*U* = 7.500	*t* = −0.555	*t* = −0.655	*t* = −0.240	*t* = −1.133	*t* = −0.533	*t* = −1.419	*t* = −0.938
	*Z* = −0.145	df = 6	df = 6	df = 6	df = 6	df = 6	df = 6	df = 6
	*P* = 0.886	*P* = 0.599	*P* = 0.537	*P* = 0.819	*P* = 0.301	*P* = 0.613	*P* = 0.206	*P* = 0.384
*F. occidentalis*								
C1	1.40 ± 0.45	1.53 ± 0.49	2.15 ± 0.13	1.50 ± 0.65	1.10 ± 0.28	1.83 ± 0.34	1.13 ± 0.24	1.30 ± 0.20
C2	1.40 ± 0.31	2.35 ± 0.58	1.43 ± 0.23	1.08 ± 0.35	0.73 ± 0.17	1.35 ± 0.10	1.05 ± 0.09	1.25 ± 0.19
	*t* = 0.000	*t* = −1.085	*t* = −2.621	*t* = −0.0.576	*U* = 5.000	*t* = −1.413	*t* = −0.193	*t* = −0.182
	df = 6	df = 6	df = 6	df = 6	*Z* = −1.155	df = 6	df = 6	df = 6
	*P* = 1.000	*P* = 0.320	*P* = 0.059	*P* = 0.585	*P* = 0.343	*P* = 0.207	*P* = 0.854	*P* = 0.862

Note: Data within the same column was analyzed through Student's *t*‐test or Mann–Whitney *U‐*test to compare homogeneity of control plots (*P* < 0.05).

**Table 3:** Statistics and interactions among factors in the tomato and sweet pepper trials according to a Linear Mixed Model using insect and pesticide as fixed factors, and sampling dates as the repeated measures factor (5 weeks in tomato and 8 weeks in sweet pepper) (*P* < 0.05)

Tomato 2016; *Nesidiocoris tenuis*, *Tuta absoluta*	
Insect	*F*_1,174.530_ = 9048.358; *P* < 0.001
Pesticide	*F*_6,174.530_ = 138.829; *P* < 0.001
Insect × Pesticide	*F*_6,174.530_ = 133.346; *P* < 0.001

Tomato 2017; *Nesidiocoris tenuis*, *Tuta absoluta*	
Insect	*F*_1,150.319_ = 5139.886; *P* < 0.001
Pesticide	*F*_5,150.319_ = 233.893; *P* < 0.001
Insect × Pesticide	*F*_5,150.319_ = 81.817; *P* < 0.001

Sweet pepper 2016; *Amblyseius swirskii*, *Orius laevigatus*, *Frankliniella occidentalis*	
Insect	*F*_2,529.434_ = 788.333; *P* < 0.001
Pesticide	*F*_5,529.434_ = 4.368; *P* = 0.001
Insect × Pesticide	*F*_10,529.434_ = 40.181; *P* < 0.001

### Nesidiocoris tenuis and T. absoluta in tomato

The pattern of weighted average numbers of *N. tenuis* was cohesive both years and significant differences among treatments accentuated with time (2016: *F*_6,94.955_ = 162.134; *P* < 0.001; 2017: *F*_5,79.356_ = 168.984; *P* < 0.001) (Fig. A and B). Control plots had the highest density of mirids (up to 6 individuals per leaf the last evaluation date, both years). Under the two diamides treatments (flubendiamide and chlorantraniliprole, the latter only used in 2016), mirid density was similar to control and increased over time even after the insecticide applications. On the last sampling date of each year, numbers of mirids under metaflumizone, emamectin benzoate and spinosad treatments were close to the initial density registered before treatments and significantly lower than those registered for the two diamides and the control. Chlorpyrifos‐methyl was the most aggressive pesticide both years and its application decreased the mirid population approximately by 35% since the starting of the trial.

**Figure 1:**
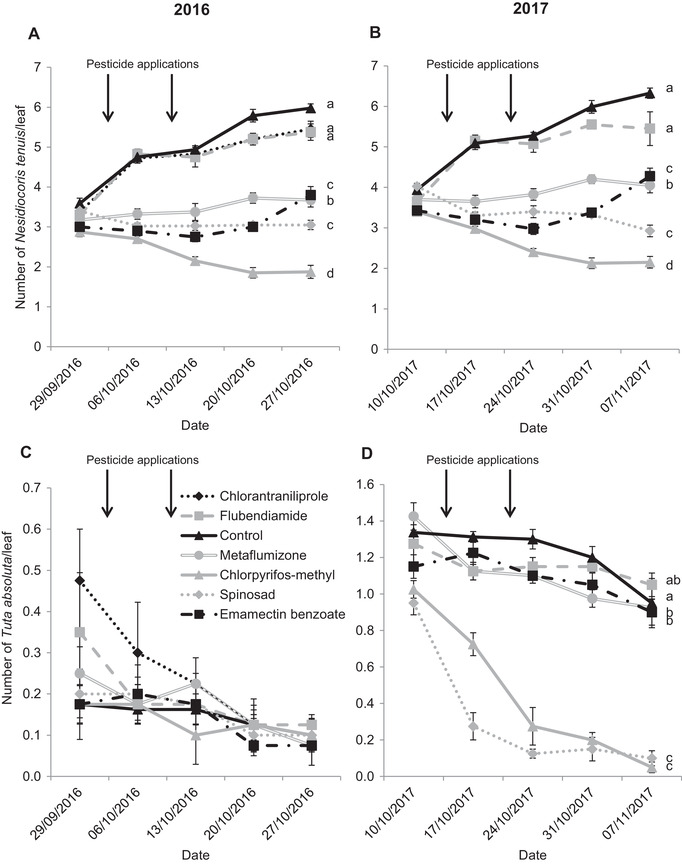
Mean ± SEM population of *Nesidiocoris tenuis* and *Tuta absoluta* per leaf inside tomato commercial greenhouses in 2016 (A and C) and 2017 (B and D) after two pesticide applications on September 30 and October 11, 2016, and on October 11 and 21, 2017. Arrows point out the moment of pesticide applications. Different letters stand for statistical differences among treatments according to a Linear Mixed Model test using pesticide as the fixed factor and sampling dates as the repeated measures factor, followed by LSD pairwise comparisons (*P* < 0.05).

The pesticide impact on *T. absoluta* population varied each year (Fig. C and D). In 2016, density was extremely low (from 0.48 ± 0.13 to 0.08 ± 0.05 individual per leaf) and no statistically significant differences were observed among treatments (*F*_6,131.379_ = 0.947; *P* = 0.464) (Fig. C). However, in 2017 the initial population density was 3‐fold higher (up to 1.43 ± 0.08 individuals per leaf; Fig. D). Chlorpyrifos‐methyl and spinosad significantly decreased population over time, which reached values near zero in the last monitoring date. Flubendiamide behaved like the control, and metaflumizone and emamectin benzoate only caused punctual significant decreases (*F*_5,74.664_ = 162.591; *P* < 0.001) (Fig. D).

### Orius laevigatus, A. swirskii, and F. occidentalis in sweet pepper

The presence of *F. occidentalis* over the different sampling dates was low and pesticide treatments were not very successfully effective (Table 4). The thrip population significantly grew in plots treated with pymetrozine, spirotetramat, sulfoxaflor, and chlorantraniliprole compared to control and emamectin benzoate (*F*_5,177.538_ = 31.549; *P* < 0.001). Both NE also settled in the crop at low densities, which decreased in all treatments over time (Table 4). Sulfoxaflor was the most toxic pesticide to *O. laevigatus* although statistically similar to pymetrozine, followed by spirotetramat and emamectin benzoate (*F*_5,178.159_ = 30.725; *P* < 0.001). Chlorantraniliprole was harmless and statistically similar to control and emamectin benzoate. For *A. swirskii*, density in control and emamectin benzoate was significantly different to rest of pesticides (*F*_5,157.977_ = 10.262; *P* < 0.001). Sulfoxaflor was slightly harmful, and emamectin benzoate, spirotetramat, pymetrozine, and chlorantraniliprole harmless, based on IOBC toxicity categories.

**Table 4:** Mean ± SEM population of *Orius laevigatus*, *Amblyseius swirskii*, and *Frankliniella occidentalis* per leaf in sweet pepper commercial greenhouses with pesticide applications on October 6 and 18, 2016

Sweet pepper 2016	Oct 5	Oct 12	Oct 19	Oct 26	Nov 3	Nov 9	Nov 16	Nov 23	
*O. laevigatus*									
Control	1.04 ± 0.15	0.93 ± 0.13	0.94 ± 0.09	1.11 ± 0.12	1.05 ± 0.14	0.74 ± 0.09	0.89 ± 0.08	0.84 ± 0.08	a
Chlorantraniliprole	1.08 ± 0.10	0.90 ± 0.07	0.95 ± 0.23	1.05 ± 0.10	1.23 ± 0.12	0.85 ± 0.13	0.73 ± 0.09	0.65 ± 0.06	ab
Emamectin benzoate	1.15 ± 0.35	1.08 ± 0.10	0.85 ± 0.12	0.90 ± 0.11	0.78 ± 0.08	0.88 ± 0.16	0.75 ± 0.10	0.55 ± 0.10	b
Spirotetramat	1.25 ± 0.10	0.93 ± 0.12	0.65 ± 0.09	0.53 ± 0.13	0.45 ± 0.03	0.43 ± 0.09	0.45 ± 0.06	0.53 ± 0.05	c
Pymetrozine	0.95 ± 0.13	1.00 ± 0.16	0.53 ± 0.07	0.33 ± 0.05	0.48 ± 0.09	0.30 ± 0.04	0.33 ± 0.05	0.53 ± 0.06	cd
Sulfoxaflor	1.33 ± 0.15	0.95 ± 0.09	0.70 ± 0.07	0.40 ± 0.04	0.48 ± 0.06	0.15 ± 0.03	0.25 ± 0.10	0.23 ± 0.08	d
*A. swirskii*									
Control	3.49 ± 0.34	3.06 ± 0.32	2.96 ± 0.16	2.41 ± 0.22	2.66 ± 0.12	2.38 ± 0.13	2.64 ± 0.20	2.84 ± 0.08	a
Chlorantraniliprole	3.35 ± 0.44	2.70 ± 0.29	1.60 ± 0.11	2.48 ± 0.32	2.33 ± 0.17	2.18 ± 0.14	2.28 ± 0.13	3.35 ± 0.17	b
Emamectin benzoate	3.93 ± 0.26	3.70 ± 0.40	2.45 ± 0.36	2.65 ± 0.26	2.53 ± 0.27	2.50 ± 0.11	2.55 ± 0.14	2.48 ± 0.11	a
Spirotetramat	3.13 ± 0.40	2.80 ± 0.34	2.05 ± 0.21	1.78 ± 0.12	1.70 ± 0.20	2.13 ± 0.09	2.15 ± 0.21	2.28 ± 0.13	b
Pymetrozine	2.93 ± 0.33	2.68 ± 0.09	2.58 ± 0.46	2.20 ± 0.44	2.00 ± 0.15	1.98 ± 0.11	2.55 ± 0.12	2.18 ± 0.11	b
Sulfoxaflor	4.03 ± 0.39	3.08 ± 0.32	1.95 ± 0.24	1.88 ± 0.13	2.10 ± 0.11	2.20 ± 0.21	2.33 ± 0.19	1.63 ± 0.26	b
*F. occidentalis*									
Control	1.40 ± 0.25	1.94 ± 0.38	1.79 ± 0.18	1.29 ± 0.35	0.91 ± 0.17	1.59 ± 0.19	1.09 ± 0.12	1.28 ± 0.13	c
Chlorantraniliprole	1.68 ± 0.13	2.18 ± 0.41	1.93 ± 0.37	1.53 ± 0.28	1.28 ± 0.22	1.85 ± 0.20	2.83 ± 0.98	2.33 ± 0.37	b
Emamectin benzoate	1.00 ± 0.07	1.05 ± 0.13	1.85 ± 0.18	1.70 ± 0.25	2.03 ± 0.43	1.73 ± 0.24	1.58 ± 0.28	1.18 ± 0.24	c
Spirotetramat	2.13 ± 0.36	2.28 ± 0.35	2.68 ± 0.39	3.73 ± 0.54	4.55 ± 0.62	4.13 ± 0.71	3.33 ± 0.48	2.48 ± 0.25	a
Pymetrozine	2.10 ± 0.18	1.95 ± 0.48	4.28 ± 0.73	4.93 ± 0.36	4.15 ± 1.03	3.65 ± 0.72	3.18 ± 0.35	2.73 ± 0.44	a
Sulfoxaflor	1.53 ± 0.22	1.55 ± 0.32	1.45 ± 0.34	4.23 ± 0.35	3.68 ± 0.70	2.83 ± 0.44	2.63 ± 0.23	2.40 ± 0.21	b

Note: Different letters for each insect stand for statistical differences among treatments throughout the whole duration of the experiment according to a Linear Mixed Model test using pesticide as the fixed factor and sampling dates as the repeated measures factor, followed by LSD pairwise comparisons (*P* < 0.05).

Final IOBC pesticide toxicity categories in both crops are shown in Table 5.

**Table 5:** Final IOBC toxicity categories† based on the mortality with two pesticide applications to the natural enemies in the commercial tomato and sweet pepper greenhouses

		*Nesidiocoris tenuis*		*Orius laevigatus*	*Amblyseius swirskii*
Pesticide	MFRC‡ (g a.i./ha)	2016	2017	2016	2016
Chlorantraniliprole	40	1	Nontested	1	1
Chlorpyrifos‐methyl	900	3	3	Nontested	Nontested
Emamectin benzoate	12.75	2	2	2	1
Flubendiamide	90	1	1	Nontested	Nontested
Metaflumizone	240	2	2	Nontested	Nontested
Spinosad	75	2	3	Nontested	Nontested
Spirotetramat	75	Nontested	Nontested	2	1
Pymetrozine	250	Nontested	Nontested	2	1
Sulfoxaflor	24	Nontested	Nontested	3	2

^†^ IOBC toxicity categories for field test: 1 = harmless (<25% mortality); 2 = slightly harmful (25%–50% mortality); 3 = moderately harmful (51%–75% mortality); and 4 = harmful (>75% mortality).

^‡^ Maximum field recommended concentration.

## Discussion

The use of NE is the key control strategy in the greenhouses of Almería. At present, omnivorous predatory species are the most recommended because pest pressure is high from the beginning of the crop cycle and NE can establish early, which is essential to their success. In our trials, the three released NE successfully established in the crops before pest arrival. *Amblyseius swirskii* can feed on other available prey species (e.g., the mite *B. hernandezi* was present in the crop), pollen and preys provided in the formulation. *Nesidiocoris tenuis* can prey exclusively on *T. absoluta* eggs and larvae (Biondi *et al*., 2018), plant sap and the alternative food provided. *Orius laevigatus* has a marked preference for thrips but the genus can also feed on pollen, xylem and mesophyll contents (Armer *et al*., 1998).

Biological control is nowadays applied in Almería in more than 50% of the total greenhouse surface (Biocolor, 2018; MAPAMA, 2018a), encouraged by pressure from supply chain and consumers (Glass & Egea González, 2012), pesticide resistance (Bielza & Gillén, 2014; Grávalos *et al*., 2014; Roditakis *et al*., 2018), and the EU legislation making IPM obligatory (OJEU, 2009). Ideally, the adoption of biological control alone in protected cultures should be possible because of the emphasis on sustainable production systems and the great deal of NE commercially available worldwide. However, the adoption can sometimes be difficult because the risk tolerance of farmers is usually very low, especially during harvest; consumers demand aesthetic products; the governments not always give support to this strategy and there is dominance of the pesticide industry (van Lenteren, 2011). Besides, crops are threatened by key and secondary pests that can coexist, invasive pest species or emergent virus diseases, as it has unfortunately happened in the region during the last decades (Robledo *et al*., 2009; Parrella & Lewis, 2018).

Therefore, there is still reliance on pesticide applications in punctual moments when NE are not sufficiently efficient. Pesticides applied can negatively affect parasitoids and predators even when releases are carefully timed (Bielza *et al*., 2009; Colomer *et al*., 2011; Gázquez *et al*., 2011; Amor *et al*., 2012); therefore, it is essential to ascertain their compatibility prior use (IOBC, 2018).

Multiple exposure routes enhance the pesticide risk to NE, as reported by Madbouni *et al*. (2017) for *N. tenuis*. In our trials, as NE were introduced in the nursery or early in the crop, possible sources of contamination are contact with residues on leaves or with droplets during application, and feeding on contaminated preys or alternative food. Multiple pesticide applications or pesticide mixtures also entail a greater risk to NE than single applications (Panizzi *et al*., 2017), but in literature there is scarce information.

In our commercial greenhouses, the harmfulness of the tested pesticides for our NE agree with results published in several pesticide databases, even though it is difficult to know exactly how these data were generated (Biobest, 2019; Koppert, 2019), except for IOBC database, where references are added (IOBC, 2019). The fungicide tebuconazole did not affect the populations of *N. tenuis*, *O. laevigatus* and *A. swirskii* in control plots, which were higher than under the majority of pesticide‐treated plots. Tebuconazole is selective for phytoseiid mites (Fountain & Med, 2015; Put *et al*., 2016), *Orius* spp. (Biobest, 2019) and other NE in semifield and field (Sterk *et al*., 1999). Besides, the fungicide seemed not to have synergized effects with any of our pesticides despite they are reported with neonicotinoid thiacloprid (Willow *et al*., 2019).

The tomato crop was only attacked by the leafminer *T. absoluta*. In the second year (2017), the initial population on the first monitoring date was threefold higher than in 2016 and decreased over time, especially with the spinosad and chlorpyrifos‐methyl treatments (89% and 95% reduction in final monitoring compared to the initial, respectively). In contrast, flubendiamide, emamectin benzoate, and metaflumizone did not control our population. This pest exhibits widespread or moderate resistance to diamides and spinosyns in several world areas (Biondi *et al*., 2018; Grant *et al*., 2019). In Spain, different populations have developed high resistance to chlorpyrifos (Haddi *et al*., 2017) and moderate to chlorantraniliprole (Roditakis *et al*., 2017, 2018), but many others have not shown any resistance to spinosad and emamectin benzoate (Roditakis *et al*., 2018). The uneven distribution of resistance in field populations may have played a role in our results.

There is little information in the literature about pesticide compatibility with *N. tenuis* under field settings. The NE, established in the tomato nurseries was not negatively affected by two applications of the diamides flubendiamide and chlorantraniliprole, and density grew up to 49%–67% at the end of the trial (IOBC category 1). The number of mirids per plant in these plots always surpassed the economic threshold of 4.5, which is reported to result in less than 4% damaged tomatoes (Arnó *et al*., 2011). One application of flubendiamide was also compatible with *N. tenuis*, released in plants as soon as residues were dried (Wanumen *et al*., 2016). Two applications of emamectin benzoate impaired the population growth of the NE (IOBC 2; 9%–25% increase in density). This result is in accordance with the long duration of emamectin benzoate harmful activity to *N. tenuis* under semifield conditions (IOBC category C in the persistence scale; Hassan, 1994) reported by Wanumen *et al*. (2016). In contrast, one application of emamectin in semifield was safe for the mirid (López *et al*., 2011). The most deleterious pesticide was chlorpyrifos‐methyl (IOBC 3; 35%–37% reduction), followed by spinosad (IOBC 2–3), the latter compatible in extended laboratory trials after one application at a lower concentration than that used in our trials (72 instead of 110 g a.i/ha) (Arnó & Gabarra, 2011).

In sweet pepper, the biological control of the thrip *F. occidentalis* relies on the anthocorid *O. laevigatus* (very effective) and the phytoseiid mite *A. swirskii* (more polyphagous but with a very fast establishment in the crop) (Robledo *et al*., 2009). In our trial, both NE established in the crop at low population levels. Levels of *F. occidentalis* were very low and not homogeneous at the beginning of the trial. Thrips slightly grew under pymetrozine, spirotetramat, and sulfoxaflor (16%–57% increase), probably because these pesticides also decreased the population of *O. laevigatus*, and maybe because of the broad insecticide resistance in Spanish populations (Bielza, 2008, 2009).

In contrast to our results, neither spirotetramat to *O. armatus* nor spiromesifen (same mode of action of spirotetramat, group 23; IRAC, 2018) to *O. laevigatus* were toxic after one application (Bielza *et al*., 2009; Broughton *et al*., 2013). Pymetrozine, reported as harmless to *O. laevigatus* in semifield (van de Veire *et al*., 2002), can decrease fecundity and nymph hatch of *O. armatus* (Broughton *et al*., 2013), and this could have played a role in our results (IOBC 2). Also, the variability in the susceptibility of *O. laevigatus* to insecticides might be explained by the populations used in each study (Balanza *et al*., 2019).

Chlorantraniliprole, very selective to *Orius* species *O. insidiosus* (Say) and *O. armatus* (Gross) (Gradish *et al*., 2011; Broughton *et al*., 2013), was compatible with *O. laevigatus* and *A. swirskii* (IOBC 1). However, thrips population increase along the trail, even though there is no information in literature concerning the development of resistance to diamide compounds in Spain (Bielza & Guillén, 2014). Emamectin benzoate, slightly harmful to *O. laevigatus* either after one direct spray field application (Amor *et al*., 2012) or after two applications in our trials (IOBC 2), did not allow the pest to increase. Probably, this pesticide properly controlled *F. occidentalis* larvae since adults are in general resistant (Shan *et al*., 2012).

The predatory mite *A. swirskii* is rather compatible with pesticides under laboratory (flubendiamide, methoxyfenozide, spiromesifen, spirotetramat, metaflumizone) (Gradish *et al*., 2011; Fernández *et al*., 2017) or field conditions (methoxyfenozide, flonicamid, emamectin benzoate) (Colomer *et al*., 2011; Amor *et al*., 2012). Sulfoxaflor was slightly toxic (IOBC 2) but pesticide formulation is pertinent for compatibility. In contrast with our results, an experimental formulation of sulfoxaflor (60 mg a.i./L, 11.4% SC; Dow Agrosciences Ibérica S.A.) was compatible with adults of this predatory mite in the laboratory (Fernández *et al*., 2017). In agreement with literature, two applications of the rest of the tested pesticides were compatible with *A. swirskii*. Chlorantraniliprole is reported harmless to the phytoseiid mite *Iphiseius degenerans* (Berlese) under laboratory and greenhouse (Gradish *et al*., 2011; Döker *et al*., 2014). A short duration of the pesticide harmful activity is important for NE safety. As such, emamectin benzoate (IOBC category A in the persistence scale; Fernández *et al*., 2017) and spirotetramat were compatible after two applications in our trial (Koppert, 2019). However, another formulation of emamectin benzoate has been rated as slightly harmful or harmful (Koppert, 2019), probably because phytoseiid community level interactions, different in every field trial, play a role (Bakker & Jacas, 1995).

To sum up, our research results herein are suitable for employment in the rational planning of IPM programmes in vegetable greenhouses. Both the mode of action of pesticides (IRAC, 2018) and the number of applications are important for the selectivity. The diamides flubendiamide and chlorantraniliprole (IRAC group 28) can be included in tomato IPM programmes because *N. tenuis* coexists perfectly with them after two applications. Emamectin benzoate and metaflumizone (IRAC 6 and 22, respectively) should be handled with care because they were slightly harmful after two applications. The organophosphate chlorpyrifos‐methyl and the spynosin spinosad (IRAC 1 and 5, respectively) reduced the low populations of *T. absoluta*, but they compromised the activity of the NE as well. In the sweet pepper crop, two applications of most of the pesticides tested, chorantraniliprole, emamectin benzoate, spirotetramat (IRAC 23) and pymetrozine (IRAC 9) were harmless to *A. swirskii* and can be recommended for IPM programmes. Only the sulfoximine sulfoxaflor (IRAC 4) was slightly harmful. *Orius laevigatus* was less tolerant to the pesticides and only chlorantraniliprole was harmless.

## Disclosure

Authors declare no conflict of interest.
